# Perceptions and beliefs about anaemia: A qualitative study in three agroecological regions of Ghana

**DOI:** 10.1111/mcn.13181

**Published:** 2021-03-29

**Authors:** Raphael Baffour Awuah, Esi K. Colecraft, Mark L. Wilson, Leonard Kofi Adjorlolo, Nathalie J. Lambrecht, Hanson Nyantakyi‐Frimpong, Andrew D. Jones

**Affiliations:** ^1^ Regional Institute for Population Studies University of Ghana Accra Ghana; ^2^ Department of Nutrition and Food Science University of Ghana Accra Ghana; ^3^ Department of Epidemiology, School of Public Health University of Michigan Ann Arbor Michigan USA; ^4^ Livestock and Poultry Research Centre, College of Basic and Applied Sciences University of Ghana Accra Ghana; ^5^ Department of Nutritional Sciences, School of Public Health University of Michigan Ann Arbor Michigan USA; ^6^ Department of Geography and the Environment University of Denver Denver Colorado USA

**Keywords:** adolescent girls, anaemia, community, knowledge, qualitative methods, women of childbearing age

## Abstract

Little evidence exists concerning perceptions of anaemia in Ghanaian communities, which limits understanding of how to potentially improve health in settings with high anaemia prevalence. We explored lay perceptions of anaemia to understand local knowledge and beliefs and to provide an opportunity to inform interventions. A cross‐sectional, qualitative study was conducted in selected communities in three regions of Ghana with high prevalence of anaemia. Forty‐eight focus group discussions (FGDs) were conducted with adolescent girls, adult women of reproductive age and adult men (16 FGDs for each demographic group). Participants across the three demographic groups generally described anaemia as inadequate blood in the body and reported that poor diet, heat, alcohol intake, physiological factors and diseases such as malaria were the main causes of anaemia. Consequences of anaemia mentioned in the FGDs included dizziness, weight loss, loss of appetite and weakness. Prevention of anaemia was perceived to result from improved diet, avoidance of exposure to heat and improved sanitation to avoid diseases. The findings suggest that despite areas of convergence between lay and biomedical knowledge on the causes, consequences and prevention of anaemia, the burden of anaemia remains high in the study regions. This highlights a disconnect between local knowledge of anaemia and the health and nutrition behaviours needed to reduce its incidence. Effective interventions can be developed with and for communities that build upon existing knowledge while filling remaining knowledge gaps or misconceptions.

**Key messages**

- Anaemia remains a ubiquitous public health problem in Ghana, but there is a paucity of evidence on community perceptions and hence a limited understanding of gaps in local knowledge.
- Community views on the causes of anaemia, although not comprehensive, are largely aligned with the common causes in low‐ and middle‐income countries (i.e., nutritional deficiencies and infectious disease).
- Lay perceptions of anaemia prevention include improved diet, avoidance of exposure to heat and improved sanitation to avoid diseases.
- Despite areas of convergence between lay and biomedical knowledge on the causes, consequences and prevention of anaemia, anaemia prevalence remains high in Ghana, highlighting a disconnect between knowledge and the health and nutrition behaviours needed to reduce its incidence.
- Effective interventions can be developed with community engagement that build upon existing knowledge while filling remaining knowledge gaps or misconceptions that can be addressed by researchers and policymakers.

## 1 INTRODUCTION

With a global prevalence of approximately 33%, anaemia is a ubiquitous public health problem—with adolescent girls and women of reproductive age (WRA) having the highest burden (Global Nutrition Report, 2020; World Health Organization [WHO], 2015). The majority of high‐risk populations are found in low‐ and middle‐income countries (Kassebaum et al., 2014).

About half of all anaemia cases are due to iron deficiency (WHO, 2008, 2015). However, a systematic analysis of nationally representative surveys on the prevalence of iron‐deficiency anaemia showed that the contribution of iron deficiency to anaemia varies by geographical region, inflammation exposure and urban/rural setting (Petry et al., 2016).

Anaemia prevalence in sub‐Saharan Africa is estimated at about 39% among WRA (WHO, 2015). In Ghana, approximately 42% of WRA have anaemia, yet the Northern, Volta and Central regions have higher prevalence at approximately 49%, 48% and 47%, respectively (Ghana Statistical Service [GSS], Ghana Health Service [GHS], & ICF International, 2015). Given efforts to control anaemia in Ghana over the last two decades, the current prevalence of anaemia among WRA is still quite high.

In 2003, the GHS developed a 5‐year integrated anaemia control strategy, which targeted pregnant women, preschool and school‐aged children with food‐based interventions and activities to control malaria and helminth infection. Beyond these interventions, there have been efforts to strengthen the antenatal care system with an integrated package of services for pregnant women (including iron–folic acid supplementation and intermittent preventive treatment of malaria), food fortification, school‐based deworming activities and community‐level engagement to prevent and control anaemia (including mother‐to‐mother support groups and water, sanitation and hygiene [WASH] initiatives) (Strengthening Partnerships, Results, and Innovations in Nutrition Globally Project [SPRING], 2017; SPRING & GHS, 2016).

These efforts have, however, yielded mixed outcomes. For example, iron–folic acid supplementation intake among pregnant women in the Northern and Central regions, respectively, increased from 12% and 37% in 2008 to 53% and 58% in 2014, respectively. Yet, in the Volta Region, there was a decrease from 52% to 39% in the same period (GSS, GHS, & ICF International, 2015; GSS, GHS, & ICF Macro, 2009). Deworming coverage among children also decreased from 50% in 2008 to 42% in 2014 in the Central Region. Yet there was a marginal increase in coverage in the Volta Region from 30% to 31% and no change in per cent coverage in the Northern Region (at 14%) in the 6‐year period (GSS, GHS, & ICF International, 2015; GSS, GHS, & ICF Macro, 2009).

Many of the studies on anaemia in Ghana have been limited to assessing prevalence and determining risk factors among pregnant women (Engmann et al., 2008; Glover‐Amengor et al., 2005; Intiful et al., 2016; Mockenhaupt et al., 2000; Stephens et al., 2014). There is a paucity of evidence on perceptions of anaemia in Ghanaian communities and hence a limited understanding of gaps in local knowledge and how this could potentially drive the burden of anaemia as well as efforts to reduce its prevalence.

Evidence suggests that lack of knowledge and/or misconceptions about anaemia may be a barrier to people's acceptance and effective participation in potentially beneficial or proven interventions. For example, in South India, negative beliefs about the effects of iron supplementation on birth outcomes (beliefs that it will make the baby dark and/or big) were a barrier to iron supplement compliance (Gowri et al., 2017). In addition, a small study in Ghana among pregnant women recruited from a community health facility showed that although 63% of them identified poor diet as a cause of anaemia, less than 20% could name anaemia‐mitigating food sources (Dwumfour‐Asare & Kwapong, 2013).

This current study explored lay perceptions of anaemia among different demographic groups in Ghana to better understand how local knowledge and beliefs of anaemia might help inform interventions. Community perceptions of disease, including the names used to describe it, are often shaped by sociocultural elements, which could influence disease prevalence and hinder or facilitate interventions (Dhabangi et al., 2019; Kahissay et al., 2017). In addition, theoretical models on health beliefs and behaviours assert that cultural rules and systems are closely linked to the burden of disease (Kleinman et al., 1978).

## 2 METHODS

### 2.1 Study design and setting

This cross‐sectional, qualitative study employed focus group discussions (FGDs) to explore community perceptions and beliefs about anaemia. This study was a component of a multidisciplinary research project aimed at understanding context‐specific pathways to reducing anaemia in Ghana. Details of the other components of the project have been described previously (Jones et al., 2018; Nyantakyi‐Frimpong et al., 2018).

Three environmentally and geographically different administrative regions of Ghana with the highest prevalence of anaemia in the most recent Demographic and Health Survey (GSS, GHS, & ICF International, 2015) were selected for the study. These were the Central Region (characterized by a coastal savannah ecology along its coastline and semideciduous forests across inland areas), the Northern Region (low‐lying and characterized by the Guinea savannah) and the Volta Region (characterized by coastal grassland, semideciduous forests and Guinea savannah/mangrove swamps) (Table 1).

**TABLE 1:** List of selected districts and their agroecological profile

Region	District	Agroecological profile of district
Central	Hemang	Semideciduous forest zone
	Gomoa East	Dry coastal savannah and moist semideciduous forest
Northern	Central Gonja	Guinea savannah zone
	Karaga	Guinea savannah zone
	East Mamprusi	Woodland savannah zone
Volta	Keta	Coastal savannah zone
	Hohoe	Forest savannah transitional zone
	Kpando	A mix of Guinea savannah woodland and semideciduous forest

### 2.2 Sampling strategy

The sampling approach aimed to select participants from diverse agroecological zones, thus maximizing variation in livelihood strategies across the sample (Patton, 2014). The selection of the districts was therefore done to reflect the main agroecological zones of the region. Two districts in the Central Region and three districts each in the Northern and Volta regions were selected. Discussions were held with officials of the GHS and the Department of Food and Agriculture (DoFA) in each district. These two institutions were considered important stakeholders because they have government‐mandated, national oversight responsibilities for health and nutrition (GHS) and agricultural (DoFA) issues, as well as a strong permanent presence in the districts. The purpose of the discussions was to introduce the goals of the project and obtain input into the selection of communities for data collection.

In each of the eight districts, two communities were selected on the basis of evidence of an elevated anaemia prevalence provided by the district GHS office, the presence of animal‐sourced food‐related livelihoods in the communities (such as herding cattle, rearing poultry and/or fisheries activities) and logistical accessibility to the communities. In each community, research assistants first informed community leaders about the study, who in turn explained this to community members through the public address system and/or traditional linguists.

A purposive sampling approach was used to recruit potential participants for the study. This was supported by community volunteers who moved from house to house. Involvement in the FGDs was based on availability and willingness to participate at the time of data collection. Study participants had to have resided in the community for at least 6 months. The target populations were adolescent girls (15–19 years old), adult WRA (20–49 years old), also referred to as adult women in this study, and adult men (20 years and older).

Evidence shows that the risk of anaemia is significantly higher during childbearing years (15–49 years) due to an increased need for iron during pregnancy and increased iron losses from menstruation (Orish et al., 2013). On the other hand, it is not uncommon to find anaemia among men, although the prevalence in men is the lowest relative to other demographic groups (WHO, 2008). In addition, in many parts of the study regions (and most of Ghana), about two thirds of households are headed by men (GSS, GHS, & ICF International, 2015); as such, decisions on household food consumption that affects the overall nutritional status of other household members are often made by men.

Individuals who agreed to participate in the group discussions converged at a convenient location determined in advance by the research assistants and community volunteers.

### 2.3 Data collection

In each of the communities, one FGD was completed with each of the three demographic groups, specifically adolescent girls (124 participants in total), adult women (135 participants in total) and adult men (141 participants in total). Each FGD was composed of 7 to 11 persons and averaged 8 persons. A total of 48 FGDs (16 FGDs for each demographic group, involving a total of 400 participants) were completed between February and March 2017. The FGDs lasted from 45 to 90 min and were facilitated by trained research assistants with local language competencies. Each FGD had a moderator and a note taker.

An FGD guide, which was partly informed by the explanatory models of illness, was used to collect the data. This framework highlights the complex processes of making sense of illness by individuals or groups in order to bridge the gap between biomedical explanations and lay knowledge and beliefs (Kleinman, 1980). Lay knowledge and conceptualization of illness are often influenced by social and cultural contexts, as well as prior experiences (Karasz, 2005).

Although anaemia status was not assessed in this current study, it was assumed that participants were familiar with anaemia, through personal experience, health facility testing or knowing someone who had anaemia, given that anaemia is endemic in these communities. This helped us to decipher concordance and discordance between community members' understanding of anaemia and biomedical explanations, which is useful in developing strategies for anaemia mitigation in these regions. The key areas of the FGD guide were (a) description of anaemia, (b) population groups vulnerable to anaemia, (c) causal theories of anaemia, (d) consequences of anaemia and (e) prevention of anaemia (see Appendix A).

Eighteen research assistants (eight females/ten males) were trained in the ethics and facilitation of FGDs. Before the start of data collection, the FGD guide was piloted in two communities in the Central Region with characteristics similar to the selected study communities. The piloting provided an opportunity to assess responses to the questions and also to give targeted training to the research assistants. All FGDs were audio recorded with permission from the participants.

### 2.4 Data processing and analysis

Trained personnel with prior experience in transcribing qualitative interviews transcribed all the recorded FGDs. Transcriptions were done with simultaneous translation from the local languages to English, following best practices in qualitative research (Helmich et al., 2017). Each transcript was quality checked by members of the project team who listened to the recorded interview and checked it against the interview transcription to ensure accuracy.

The transcripts were analysed after data collection was completed using the thematic analysis approach described by Attride‐Stirling (2001). Data were analysed by six project team members beginning with a discussion and the generation of an a priori list of organizing themes based on the research objectives and conceptual framework of the project. Each project team member read all the transcripts and coded them independently. The coding was guided by the initial list of organizing themes (deductive), which were modified and expanded on the basis of information derived from the transcripts (inductive). After the transcripts had been read individually, there was a group discussion on the independently generated codes to arrive at a consensus on codes that were markedly different. The next stage of the analysis involved the development of the basic coding frame (coding matrix), which provided information on the frequency of codes. Differences and commonalities in perceptions and beliefs across the different demographic groups are presented in Section 3. Representative quotes, selected by the project team, that best capture shared ideas are presented for illustration. The analysis was facilitated by the qualitative software package, Atlas.ti (Version 7.5) (Scientific Software Development GmbH).

### 2.5 Ethical considerations

The study was approved by the University of Ghana College of Basic and Applied Sciences Institutional Review Board (ECBAS 001/16‐17), the Ghana Health Service Ethical Review Committee (GHS‐ERC 07/01/17) and the University of Michigan Health Sciences and Behavioural Sciences Institutional Review Board (HUM00120734). Written informed consent was obtained from all participants.

## 3 RESULTS

### 3.1 Selected sociodemographic characteristics of the FGD participants

Table 2 shows selected sociodemographic characteristics of the study participants. In all three regions, the majority of participants in the FGDs with adolescent girls (15–19 years old) were students. About one third of the adolescent girls in the Volta Region reported that they were either married or in a union compared with almost none in the Central and Northern regions.

**TABLE 2:** Selected sociodemographic characteristics of FGD participants

Characteristic	Central Region	Volta Region	Northern Region
	*n* (%)	*n* (%)	*n* (%)
**Adolescent girls**	24 (100)	46 (100)	54 (100)
*Education*			
No education	0	1 (2.2)	8 (14.8)
Primary	0	6 (13)	14 (25.9)
JHS	23 (95.8)	35 (76.1)	12 (22.2)
SHS	1 (4.2)	4 (8.7)	20 (37.1)
*Marital status*			
Not married	24 (100)	31 (67.4)	52 (96.3)
Married/in union	0	15 (32.6)	2 (3.7)
*Occupation*			
Student	24 (100)	31 (67.4)	37 (68.5)
Artisan	0	8 (17.4)	3 (5.6)
Farmer	0	5 (10.9)	0
No occupation	0	2 (4.3)	14 (25.9)
**Adult WRA**	29 (100)	54 (100)	52 (100)
*Education*			
No education	8 (27.6)	13 (24.1)	40 (76.9)
Primary	9 (31.1)	18 (33.3)	6 (11.5)
JHS	11 (37.9)	20 (37)	3 (5.8)
SHS	1 (3.4)	3 (5.6)	3 (5.8)
*Marital status*			
Not married	2 (6.9)	1 (1.9)	3 (5.8)
Married/in union	25 (86.2)	52 (96.3)	49 (94.2)
Divorced/widowed	2 (6.9)	1 (1.9)	0
*Occupation*			
Trader	11 (47.8)	6 (11.1)	31 (59.6)
Farmer	8 (27.6)	28 (51.9)	19 (36.6)
Fisherman/fishmonger	4 (17.4)	17 (31.5)	0
Artisan	3 (10.3)	1 (1.9)	1 (1.9)
No occupation	3 (10.3)	2 (3.6)	1 (1.9)
**Men**	29 (100)	55 (100)	62 (100)
*Education*			
No education	5 (20.8)	14 (25.5)	43 (69.4)
Primary	1 (4.2)	6 (10.8)	3 (4.8)
JHS	14 (58.3)	20 (36.4)	7 (11.3)
SHS	4 (16.7)	14 (25.5)	7 (11.3)
Tertiary	0	1 (1.8)	2 (3.2)
*Marital status*			
Not married	3 (12.5)	10 (18.2)	12 (19.4)
Married/in union	21 (87.5)	45 (81.8)	50 (80.6)
*Occupation*			
Student	0	1 (1.8)	2 (3.2)
Trader	2 (8.3)	0	0
Farmer	9 (37.5)	27 (49.1)	59 (95.2)
Fisherman	5 (20.8)	20 (36.4)	0
Artisan (mason, carpenter)	7 (29.2)	5 (9.1)	0
Professional (health/teacher)	1 (4.2)	2 (3.6)	0
No occupation	0	0	1 (1.6)

Abbreviations: FGD, focus group discussion; JHS, junior high school, SHS, senior high school; WRA, women of reproductive age.

Among participants in the FGDs with adult women (20–49 years old), about three quarters from the Northern Region had no formal education, compared with roughly one quarter in the Central and Volta regions. Nearly all adult women in each of the three regions were in union, and the most common occupation was petty trading (Central and Northern regions) or farming (Volta Region).

In the FGDs with men (20 or more years old), approximately two thirds of the participants in the Northern Region had no education compared with about one quarter in the other two regions. In all three regions, participants most often reported that they were farmers, but other occupations were also represented.

### 3.2 Perceptions of anaemia

The issues emanating from the different FGDs undertaken across the three regions centred around the following four key themes: (a) descriptions/definitions of anaemia, (b) perceived causes of anaemia, (c) consequences of anaemia and (d) prevention of anaemia. Illustrative expressions of these key themes by participants are described below.

#### 3.2.1 Descriptions of anaemia

In the FGDs across the three demographic groups, anaemia was defined as having insufficient or inadequate blood in the body. Participants in the FGDs with adolescent girls, adult women and men defined anaemia similarly:

Anaemia happens when there is insufficient blood in your body. (Adolescent girls FGD, Central Gonja District, Northern Region)

Anaemia is when you do not have enough blood. (Adult women FGD, Hemang District, Central Region)

The understanding concerning anaemia is that it leads to shortage of or inadequate blood in the body. (Adult men FGD, Kpando District, Volta Region)

However, in one of the adolescent girls FGD, anaemia was described as a disease that could be transmitted sexually:

Sex disease; it (anaemia) is a sexually transmitted disease. (Adolescent girls FGD, Central Gonja District, Northern Region)

#### 3.2.2 Perceived causes of anaemia

Participants mentioned five main causes of anaemia, namely, diet, exposure to heat, alcohol intake, physiological factors and diseases.

3.2.2.1

In 39 FGDs, participants implicated dietary factors as a cause of anaemia. Although participants across the three demographic groups attributed anaemia to a generally poor diet, in the FGDs with adolescent girls and adult women, there was an elaboration on specific aspects of diet they believed cause anaemia. For example, in one of the FGDs with adolescent girls in the Central Region, it was indicated that anaemia results from not consuming a balanced diet:

Anaemia can be caused by the lack of intake of balanced diets which contain the necessary nutrients capable of maintaining the right amount of blood in the body. (Adolescent girls FGD, Gomoa District, Central Region)

Even more specifically, it was mentioned in one of the FGDs with adult women that a predominantly carbohydrate‐based diet could lead to anaemia:

When you only eat carbohydrates, you can get anaemia. (Adult women FGD, Hemang District, Central Region)

3.2.2.2

In 15 FGDs across all the demographic groups, exposure to heat (from the sun or fire) was mentioned as a cause of anaemia. For example, in one of the FGDs with adolescent girls, one participant explained:

Exposure to heat or sitting in the sun for long hours during working hours coupled with tedious work can lead to the reduction of blood in one's body. (Adolescent girls FGD, Keta District, Volta Region)

Additionally, in five FGDs, charcoal burning was identified as a cause of anaemia. In one of the FGDs with men, the following was said:

Charcoal burning can make a woman become short of blood. (Adult men FGD, Karaga District, Northern Region)

3.2.2.3

Alcohol consumption was mentioned as a cause of anaemia in six FGDs involving adolescent girls and men in the Central and Volta regions. In one of the FGDs with adolescent girls in the Volta Region, a participant suggested that excessive intake of alcohol can cause anaemia. In another FGD with men, there was an admission that alcohol consumption is on the increase, suggesting that this has led to the increase in anaemia cases:

If you are a drunkard, it makes the blood in your body cease to flow and not work properly. Hence the shortage of blood. (Adolescent girls FGD, Hohoe District, Volta Region)

Our forefathers were not getting anaemia, but for us, we drink alcohol a lot; that is why we are getting anaemia in our bodies. (Adult men FGD, Keta District, Volta Region)

3.2.2.4

Physiological factors perceived as causes of anaemia included abortions, pregnancy and menstruation. In two FGDs (one with adolescent girls and the other with men), it was indicated that losing blood during an abortion procedure causes anaemia. In these discussions, abortions were specifically linked to adolescents:

If an adolescent girl attempts to abort a foetus and she is not careful to put measures in place to stop the blood flow, she could bleed out and this can cause anaemia. (Adolescent girls FGD, Gomoa District, Central Region)

In one of the FGDs with adult men in the Northern Region, it was indicated that pregnancy could lead to anaemia because a fetus gets its blood from the expectant mother:

The foetus depends on the mother's blood for its own blood. So the mother loses some of her blood to the foetus. (Adult men FGD, Central Gonja District, Northern Region)

Also, menstruation was mentioned in two FGDs with adolescent girls and one FGD with adult women. In these discussions, it was indicated that menstrual blood loss causes anaemia, especially among younger females. In one such discussion, a participant said:

Adolescent girls from the ages of 13 upwards can become anaemic when they lose blood during the time of the month. (Adolescent girls FGD, Gomoa District, Central Region)

3.2.2.5

Across the three demographic groups, anaemia was linked with diseases such as malaria (emerged from seven FGDs), worm infestation (emerged from three FGDs), sickle cell (emerged from one FGD) and human immunodeficiency virus (HIV) (emerged from one FGD):

Both malaria and worm infection can lead to anaemia. (Adult men FGD, Karaga District, Northern Region)

If you have sickle cell, you can get anaemia. (Adolescent girls FGD, Hemang District, Central Region)

We have sicknesses like HIV that can make you lose blood and become anaemic. (Adult women FGD, Hemang District, Central Region)

#### 3.2.3 Consequences of anaemia

Dizziness was the most common consequence of anaemia mentioned (in 16 FGDs). In one of the FGDs with men in the Central Region, it was indicated that anaemia leads to frequent dizziness and this affects livelihoods:

When you are anaemic, you develop frequent dizziness which can make you unbalanced (stagger) when you go fishing. (Adult men FGD, Gomoa District, Central Region)

Weight loss or thinness was also noted as a consequence of anaemia in 13 FGDs but more commonly mentioned in the FGDs with adolescent girls. A participant in one of the FGDs with adolescent girls said this:

The anaemic person may also lose weight as a result. (Adolescent girls FGD, Central Gonja District, Northern Region)

Other consequences of anaemia mentioned, particularly in the FGDs with adolescent girls and men, were weakness (emerged from 11 FGDs) and loss of appetite (emerged from five FGDs):

Anaemia can result in body weakness. This is a very common occurrence if you have anaemia (Adolescent girls FGD, Karaga District, Northern Region)

When you have anaemia, you cannot eat properly; when you are offered food even when you are hungry, you cannot eat it, which means you cannot work effectively. (Adult men FGD, Hemang District, Central Region)

#### 3.2.4 Prevention of anaemia

An improved diet was the most commonly mentioned mechanism for preventing anaemia (in 38 FGDs). Specific aspects of diet were mentioned in the FGDs with adult women and adolescent girls. For example, in two of the group discussions with adult women, consumption of cocoyam (taro) leaves and beans were mentioned as food groups that could prevent anaemia:

You should eat foods that can increase your blood levels like kontomire (prepared from cocoyam leaves), beans and others so that it will help you to get more blood. The nurses educate us on how to eat properly, to eat healthy foods and how to take care of our bodies in spite of the arduous task of smoking fish. After this education, it is your responsibility to follow their instructions so you can get better. (Adult women FGD, Hemang and Gomoa districts, Central Region)

Also, avoiding heat exposure, from either the sun or fire, was one of the preventive strategies of anaemia mentioned in four FGDs with adult women and adolescent girls:

Anaemia can be prevented when people stop walking under the sun. (Adolescent girls FGD, Central Gonja District, Northern Region)

To avoid anaemia, we should not always be around fire. (Adult women FGD, Hemang District, Central Region)

Reducing intake of alcohol was mentioned as one of the ways to prevent anaemia in one of the FGDs with adolescent girls in the Central Region. In the same FGD, avoiding abortions was mentioned as a way of preventing blood loss, which could lead to anaemia:

For a person to prevent anaemia, the person should reduce the amount of alcohol intake. (Adolescent girls FGD, Hemang District, Central Region)

The prevention of abortions will control the excessive loss of blood in women that causes anaemia. (Adolescent girls FGD, Hemang District, Central Region)

In seven FGDs across the three demographic groups, improved hygiene or sanitary conditions were mentioned as one of the ways to prevent anaemia. In all of these discussions, participants noted that maintaining or practising good hygiene was linked to the prevention of infectious diseases, which eventually leads to the prevention of anaemia:

Practicing proper hygiene, for example, covering your food and washing of hands including the bowls, can prevent anaemia. (Adult women FGD, Central Gonja District, Northern Region)

Other ways of preventing anaemia mentioned only in the FGDs with adult women were improved livestock production leading to the consumption of animal‐sourced foods (emerged from one FGD) and deworming (emerged from one FGD):

We need assistance to improve our livestock production so that we can consume them and prevent anaemia. (Adult women FGD, Central Gonja District, Northern Region)

It is also very important to deworm timely in order to avoid being anaemic through worm infestation. (Adult women FGD, Keta District, Volta Region)

## 4 DISCUSSION

This study explored lay understanding of anaemia, including how it is described, its causes, consequences and prevention, among community members living in three regions in Ghana with high anaemia prevalence. The preponderance of opinions expressed by the participants about anaemia suggests a reasonably high level of awareness and understanding of the condition in the study communities. Anaemia was correctly described by many as a disease caused by insufficient red blood cell production, characterized as ‘insufficient or inadequate blood’ in laymen's terms. In one group discussion, however, anaemia was misunderstood as a sexually transmitted disease.

Perceptions implicating ‘poor diet’ as a cause of anaemia showed a recognition of an association between consuming a simple, uniform diet and anaemia. In addition, the reference made by some adult women to a carbohydrate‐based diet as a cause of anaemia suggests an appreciation that diets based heavily on staple foods can increase anaemia risk. Poor dietary quality is an established factor in the aetiology of anaemia (Chaparro & Suchdev, 2019). The perception that certain foods, such as carbohydrates, are insufficient to meet dietary needs aligns with the biomedical knowledge that consumption of specific food groups high in nutrients needed for red blood cell production (that is, iron, vitamin A, vitamin B_12_, folate and riboflavin) is important for preventing anaemia (Fishman et al., 2000; Santoyo‐Sánchez et al., 2015).

Several FGD participants stated that excessive alcohol consumption can cause anaemia. Findings from studies that have assessed the association between alcohol and anaemia are mixed. Mild to moderate alcohol consumption may actually increase iron status or reduce the prevalence of iron‐deficiency anaemia (Looker et al., 1997; Whitfield et al., 2001), possibly through increased gastric acid secretion, iron solubilization and absorption (Malenganisho et al., 2007). Heavy alcohol use, on the other hand, may lead to gastrointestinal blood loss and interfere with red blood cell production via nutrient deficiencies such as folic acid, which can lead to anaemia (Ioannou et al., 2004). Yet heavy alcohol use has also been associated with haemochromatosis (i.e., iron overload) and lower prevalence of iron‐deficiency anaemia, possibly by increasing dietary iron absorption or through high consumption of iron‐rich alcoholic beverages (Ioannou et al., 2004). Although alcohol intake is not a primary cause of anaemia, community members' perception that excessive alcohol intake causes anaemia may reflect anecdotal experiences or a culturally developed blaming of alcohol intake on broader disease occurrence.

Perceptions of FGD participants that exposure to heat from the sun or fire can cause anaemia have also been reported in a similar study in neighbouring Cote d'Ivoire (M'Bra et al., 2013). Although participants in the present study did not specify how heat exposure leads to anaemia, participants in the Cote d'Ivoire study explained that being near fire for long periods of time can cause blood to coagulate, similar to what happens when blood in animal flesh is cooked. The participants in this study also linked charcoal burning with anaemia but did not elucidate whether the heat or smoke associated with this practice causes anaemia. At present, there is no known scientific evidence that exposure to heat causes anaemia. Exposure to smoke from biofuel, on the other hand, has been associated with anaemia among pregnant women and children, likely resulting from the effects of systemic inflammation (Kyu et al., 2010; Page et al., 2015). Such effects can compound the consequences of physiological changes during pregnancy (Page et al., 2015).

Other perceived causes of anaemia such as pregnancy, menstruation, sickle cell, malaria and worm infestation mentioned by FGD participants are consistent with known biomedical causes of anaemia (Bhutta et al., 2008; Korenromp et al., 2004; Smith & Brooker, 2010; Tolentino & Friedman, 2007). Other scientifically recognized common causes of anaemia in low‐ and middle‐income countries include nutritional deficiencies, infectious disease and genetic haemoglobin disorders (Chaparro & Suchdev, 2019). Participants' understanding of these causes of anaemia also largely aligned with some of these broad categories.

Lay perceptions of the consequences of anaemia also are consistent with the scientific pathophysiology of anaemia (e.g., fatigue, dizziness, weakness, pallor and low productivity) (Balarajan et al., 2011; Haas & Brownlie, 2001; Silverberg et al., 2001). However, other well‐established and common consequences of anaemia among pregnant women, such as increased risk of maternal mortality, miscarriage and low birth weight (Kozuki et al., 2012; Steer, 2000; Zhang et al., 2009), were not mentioned in any of the group discussions. This suggests that although knowledge on consequences may be reasonably high, it is not comprehensive.

Knowledge of anaemia prevention was related to the causes mentioned in the FGDs. Generally, the FGDs with adult women elucidated more on how anaemia could be prevented. For example, in relation to diets, the consumption of animal‐sourced foods was indicated as one of the ways to prevent anaemia. Evidence suggests that dietary diversity is associated with lower anaemia risk (Delil et al., 2018). In addition, the consumption of vegetables and legumes was mentioned as one of the pathways to preventing anaemia or reducing its incidence. Evidence shows that diets consisting of vegetables and legumes are associated with reduced anaemia (Stuetz et al., 2019).

Participants' understanding that improved hygiene can alleviate anaemia reflects the knowledge of infectious diseases as a cause of anaemia. Several infections that relate to hygiene, sanitation and safe water are significant contributors to anaemia, in addition to iron and micronutrient deficiencies (WHO, 2000). There is, however, no evidence to support participants' perceptions of avoiding heat exposure and reducing alcohol intake as anaemia prevention mechanisms.

Importantly, adult women and adolescent girls, in contrast to men, had more correct knowledge of the consequences and prevention of anaemia. Adult women, in particular, were more knowledgeable about preventive mechanisms of anaemia. This may be because women have more exposure to anaemia information through antenatal services or their own experiences with anaemia. Evidence shows that women with more knowledge of anaemia have children with lower odds of anaemia (Bilenko et al., 2007). For adolescent girls, exposure to health information in schools may contribute to their knowledge of anaemia. Thus, interventions that include health education should especially target men. This is important because anaemia among men is not inconsequential (Didzun et al., 2019) and can lead to difficulty in concentrating, fatigue and lethargy, which do not only reduce quality of life but also decrease economic productivity (Horton & Ross, 2003) and affect social relationships. Furthermore, targeting men in health education interventions is important as they often make key household decisions, such as those involving family diet, which in turn affect the health and well‐being of other household members.

As with many similar studies, a limitation of this investigation was potential bias in selection of participants for the FGDs, perhaps due to the purposive sampling approach. In addition, it is possible that some FGD participants may have been hesitant to express thoughts that were different from other group members. People also tend to feel shy in talking about health‐related issues in group‐based discussions. Despite these limitations, the concordance of the responses across the various FGD categories suggests that our findings are generally robust and provide important insights for the development of community‐specific interventions that can be scaled up to the regional or national level.

## 5 CONCLUSION

The findings suggest that perceptions of anaemia in relation to causes, consequences and prevention are largely aligned with biomedical models of anaemia. Despite areas of convergence between lay and biomedical knowledge on the causes, consequences and prevention of anaemia, evidence from a previous national study has shown that anaemia prevalence remains high in the study regions and throughout Ghana (GSS, GHS, & ICF International, 2015). This suggests that there is a disconnect between knowledge of anaemia, and the health and nutrition behaviours needed to reduce its incidence. This disconnect highlights that perceptions about the aetiology of anaemia are also complex and that a complex cluster of interventions are needed to address anaemia. To bridge this disconnect, complementary resources such as disease prevention, as well as improved health and food systems, will be required. Additional research may also be needed to understand barriers to improving health and nutrition behaviours. However, it is important to recognize that although knowledge may be necessary, it is not sufficient for behaviour change, which leads to improved health outcomes (Perumal et al., 2013). Notwithstanding, effective interventions can be developed with community engagement that build upon existing knowledge while filling remaining knowledge gaps or misconceptions that can be addressed by researchers and policymakers.

## CONFLICTS OF INTEREST

The authors declare that they have no conflicts of interest.

## CONTRIBUTIONS

RBA, ADJ and EKC conceptualized this study. RBA drafted the initial manuscript. RBA, EKC, MLW, LKA, NJL, HN‐F and ADJ contributed to the final version of the background, methods, results, discussion and conclusions. All authors read and approved the final manuscript.

## Data Availability

The data that support the findings of this study are available from the corresponding author upon reasonable request.

## Appendix A FOCUS GROUP DISCUSSION GUIDE FOR ADOLESCENT GIRLS, OTHER WOMEN OF REPRODUCTIVE AGE AND MEN

Thank you for taking the time to participate in this focus group discussion. Anaemia is a major problem particularly among adolescent girls and women, and the objective of this study is to get a better understanding community perceptions and knowledge to help identify possible solutions for reducing anaemia. There is no right or wrong answer to the questions that will be asked, so everyone should feel free to say what they know or think. We expect the discussion to last for about an hour.

1. Can you describe what anaemia is?
2. Probe: How is anaemia understood in the community?
3. Who is most likely to get anaemia?
4. Probe: Young children, adolescent girls, women, men etc.?
5. In your view, how does someone get anaemia?
6. Alternatively: What do you think causes anaemia?
7. What are the consequences of having anaemia?
8. What can be done to prevent anaemia?
